# Bow and lean test reconsidered: anatomy, predictions, and a data visualization method for horizontal canal BPPV

**DOI:** 10.1007/s00415-025-13450-0

**Published:** 2026-01-13

**Authors:** Marcello Cherchi

**Affiliations:** Department of Neurology, The University of Chicago, 5841 South Maryland Avenue, Chicago, IL 60637 USA

**Keywords:** Benign paroxysmal positional vertigo, Vestibular system, Labyrinth, Anatomy, Modeling, Treatment

## Abstract

In the diagnosis of horizontal canal benign paroxysmal positional vertigo, it is challenging to lateralize the affected side. The “bow and lean test,” and several methods built upon it, have been proposed. Many of these techniques are based on a model in which the horizontal canal’s putative anatomy involves an anteromedial segment. The actual anatomy has a posteromedial segment. We show that predictions of a model based on the hypothetical anatomy differ from those based on the true anatomy. We further show that the true anatomical model predicts that for a roll maneuver that begins in the supine position, going from the side-lying position to the prone position should generate different patterns of nystagmus depending on which side is affected; this should distinguish which side is affected, and thus has implications for treatment. We conclude with a data visualization method that depicts these findings compactly.

## Introduction

In the diagnosis of horizontal canal benign paroxysmal positional vertigo, it is challenging to lateralize the affected side. The “bow and lean test,” and several methods built upon it, have been proposed. Many of these techniques are based on a model in which the horizontal canal’s putative anatomy involves an anteromedial segment. The actual anatomy has a posteromedial segment. We discuss the different predictions resulting from these different anatomies. We further discuss the lateralizing ability predicted by the model based on true anatomy. We conclude with a data visualization method that compactly depicts these findings.

## The bow and lean test, and related methods

For horizontal canal benign paroxysmal positional vertigo (BPPV), it is challenging to determine the affected side [13], so considerable effort has been devoted to this problem. The “bow and lean test” proposed by Choung and colleagues [4] makes the following predictions:

“If the otolith lies on the right side of HSC [horizontal semicircular canal] (canalolithiasis type), the bowing nystagmus beating toward the right side (same direction as the affected ear) and the leaning nystagmus beating toward the left side was shown in BLT [bow and lean test]. Left HSC-BPPV (canalolithiasis type) shows the bowing nystagmus beating toward the left side and the leaning nystagmus beating toward the right side” [4].

According to this model, in right-sided horizontal canal BPPV, bow (tilt forward) elicits right beat (ipsiversive) nystagmus, and lean (tilt backward) elicits left beat (contraversive nystagmus); in left-sided horizontal canal BPPV, bow (tilt forward) elicits left beat (ipsiversive) nystagmus, and lean (tilt backward) elicits right beat (contraversive) nystagmus. The predictions of the model by Choung and colleagues are based on the anatomical assumption that the arc of the horizontal semicircular canal subtends an angle of only 180° (of the theoretical full circle).

**Fig. 1:**
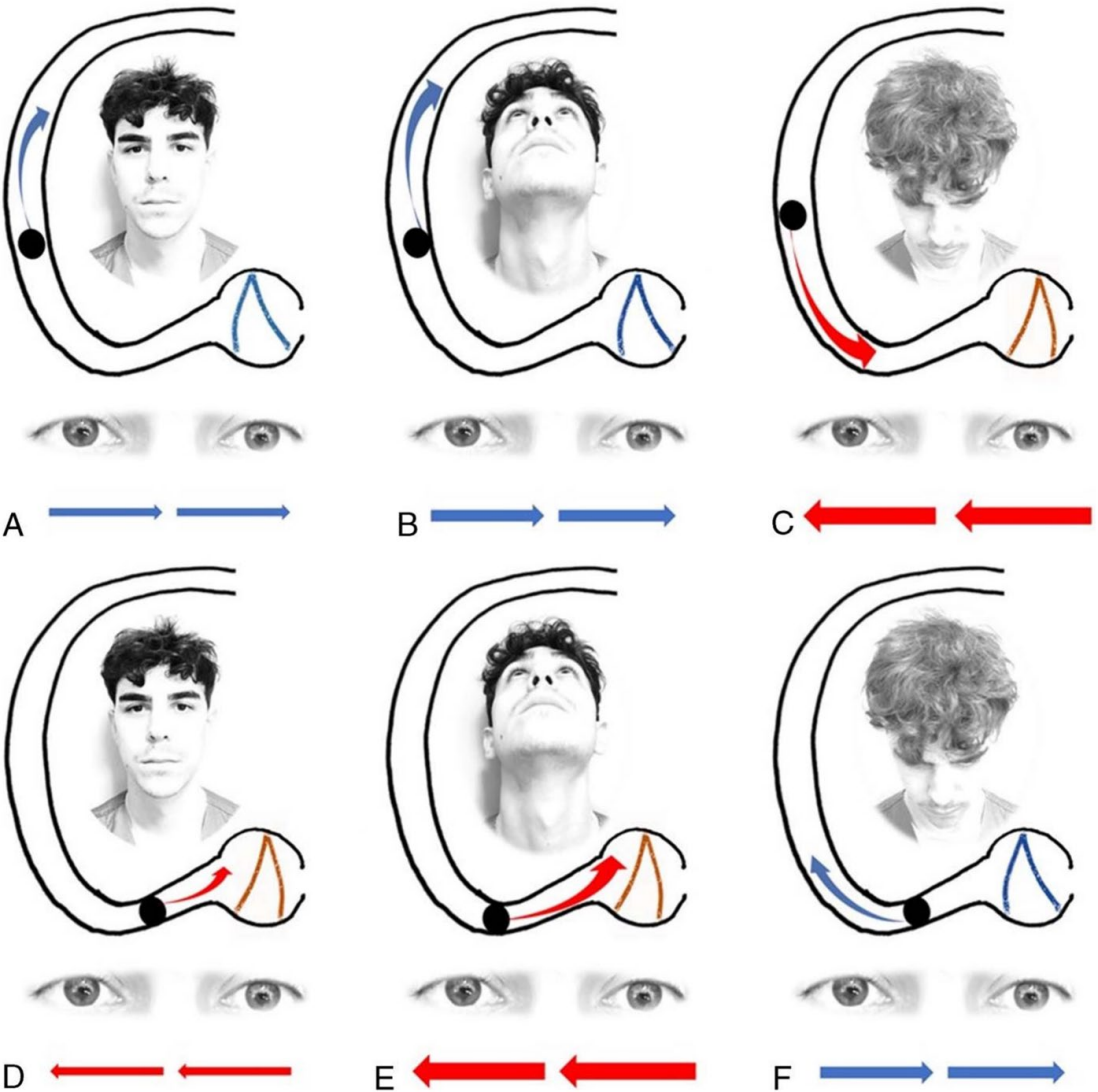
Depiction of the right-sided horizontal semicircular canal whose putative anatomy includes an anteromedial segment, from Califano et al. [3]. This model makes the following predictions. Panel **A** the canalith is in the lateral segment of the canal; when the head is in a neutral position, the horizontal semicircular canal is tilted about 30° backward, so gravity pulls the canalith posteriorly and the ampullofugal motion provokes a modest inhibitory stimulus, generating modest left beat nystagmus. Panel **B** the canalith is in the lateral segment of the canal; when the head is tilted backward, the horizontal semicircular canal is tilted nearly vertically (with the ampulla superior to the utricle), so gravity pulls the canalith posteriorly and the ampullofugal motion provokes a stronger inhibitory stimulus, generating more robust left beat nystagmus. Panel **C** the canalith is in the lateral segment of the canal; when the head is tilted forward, the horizontal semicircular canal is oriented such that the utricle is above the ampulla, so gravity pulls the canalith anteriorly, and the ampullopetal motion provokes a strong excitatory stimulus, generating robust right beat nystagmus. Panel **D** the canalith is in the anteromedial segment; when the head is in a neutral position, the horizontal semicircular canal is tilted about 30° backward, so gravity pulls the canalith posteriorly and the ampullopetal motion provokes a modest excitatory stimulus, generating modest right beat nystagmus. Panel **E** the canalith is in the anteromedial segment of the canal; when the head is tilted backward, the horizontal semicircular canal is tilted nearly vertically (with the anterior-most segment of the canal above the ampulla), so gravity pulls the canalith posteriorly and the ampullopetal motion provokes a robust excitatory stimulus, generating robust right beat nystagmus. Panel **F** the canalith is in the anteromedial segment; when the head is tilted forward, the horizontal semicircular canal is oriented such that the ampulla is above the anterior-most segment of the canal, so gravity pulls the canalith anteriorly, and the ampullofugal motion provokes an inhibitory stimulus, generating left beat nystagmus. Reproduced with written permission from Wolters Kluwer Health Limited

Several techniques, such as the “minimum stimulus” method and the “upright BPPV protocol” method, expanded on that model, but were more sophisticated in recognizing that the arc of the horizontal semicircular canal subtends an angle of about 240° (of the theoretical full circle); the “extra” 60° comes from a putative anteromedial segment. An example of this putative anatomy, and its predictions of nystagmus from the “bow and lean” test, is shown in Fig. 1, from Califano and colleagues [3].

A considerable body of literature [1–3, 10–12] uses this model which is based on a putative anatomical configuration in which the horizontal semicircular canal has an *anteromedial* segment. Yet some analyses suggest that in practice these models’ success in correctly lateralizing the affected horizontal canal is approximately equal to chance [8].

## Anatomical considerations and their predictions

**Fig. 2:**
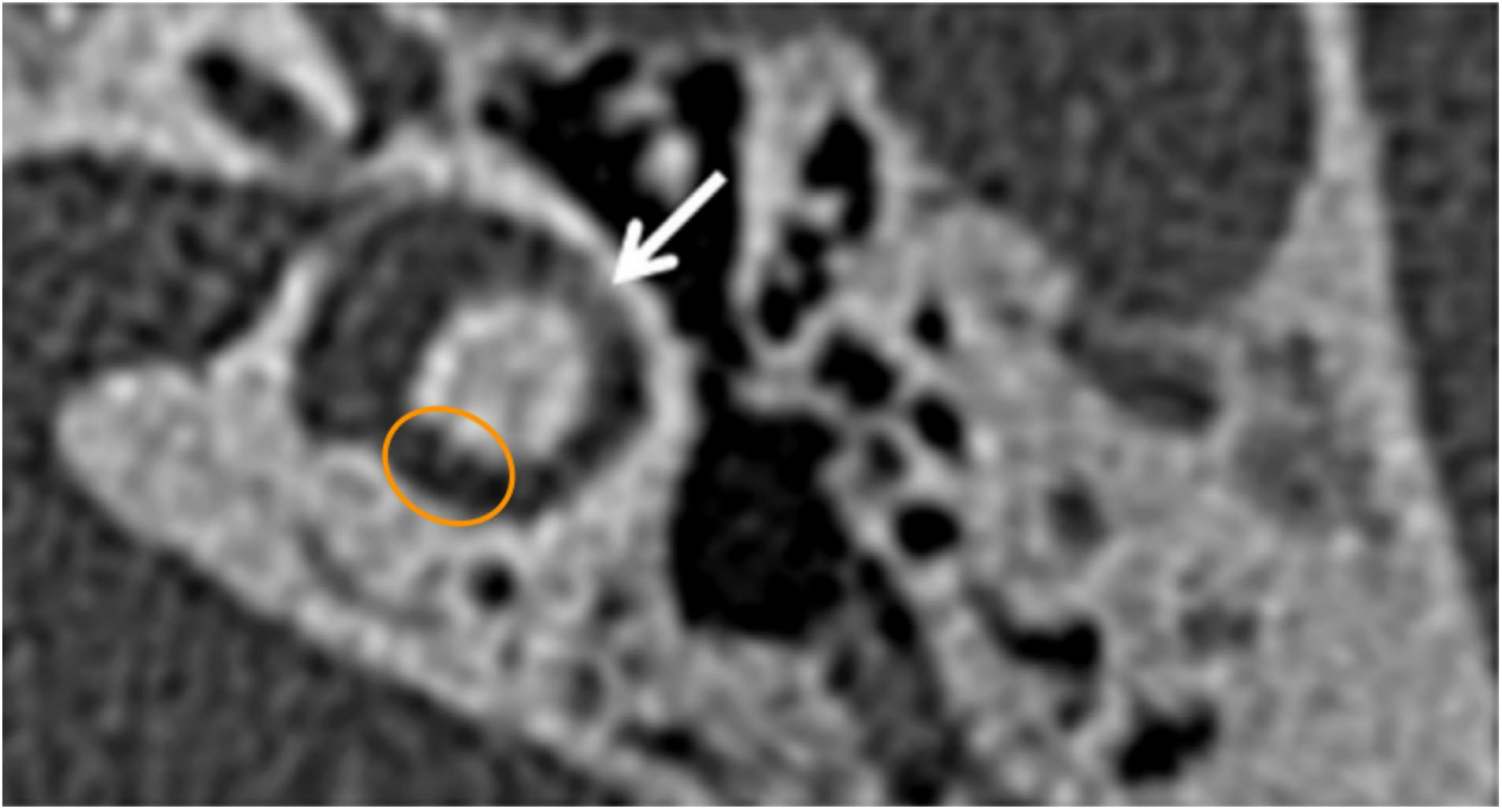
High resolution temporal bone CT of a normal left-sided horizontal semicircular canal. By convention in radiologic images, the top of the image is anterior, the bottom of the image is posterior, the left side of the image is the patient’s right, and the right side of the image is the patient’s left. The white arrow in the original image is merely indicating the horizontal semicircular canal. The orange ellipse highlights the posteromedial segment of the canal. From Oonk et al. [14]. Reproduced with written permission from Elsevier B.V.

**Fig. 3:**
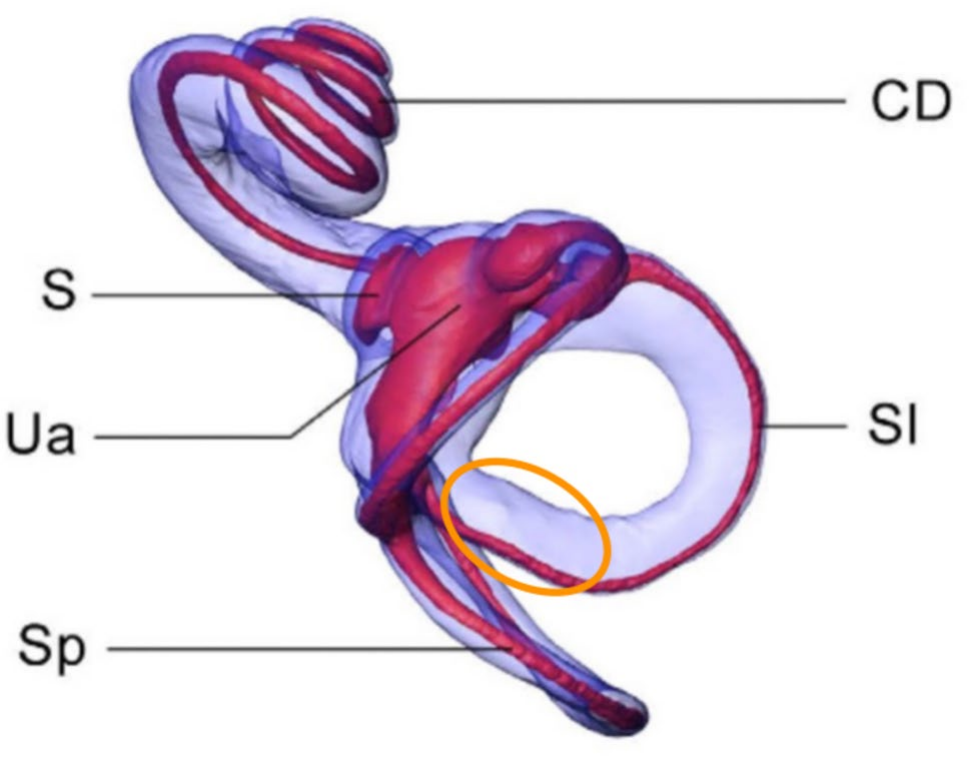
Human labyrinth reconstructed from imaging. This is the right-sided labyrinth as viewed from above; the top of the figure is anterior, the bottom of the figure is posterior, the left side of the figure is medial, the right side of the figure is lateral. *S* saccule, *Ua* anterior utricle, *Sp* slender portion of the posterior semicircular canal, *Sl* slender portion of the lateral [horizontal] semicircular canal, *CD* = cochlear duct. The orange ellipse highlights the posteromedial segment of the canal. From David et al. [5]. Reproduced with permission under CC BY 4.0

Variability in semicircular canal anatomy has been recognized for decades [7] and carefully quantified [6]. Nevertheless, even when one takes such variability into account, it turns out that the horizontal semicircular canal actually has a *posteromedial* component (not an anteromedial one), as shown in Figs. 2 and 3.

The horizontal canal’s true anatomy (with a posteromedial segment) and hypothetical anatomy (with an anteromedial segment) make different predictions about the nystagmus resulting from a logroll maneuver [9], though a meticulous step-by-step analysis is required to demonstrate this. For the situation in which otoliths are in the right-sided horizontal canal, we compare the results from rightward rotation (in Table 1) and leftward rotation (in Table 2). The human figures are generated from the publicly available website, https://posemy.art (accessed 10/2/25).

**Table 1:**
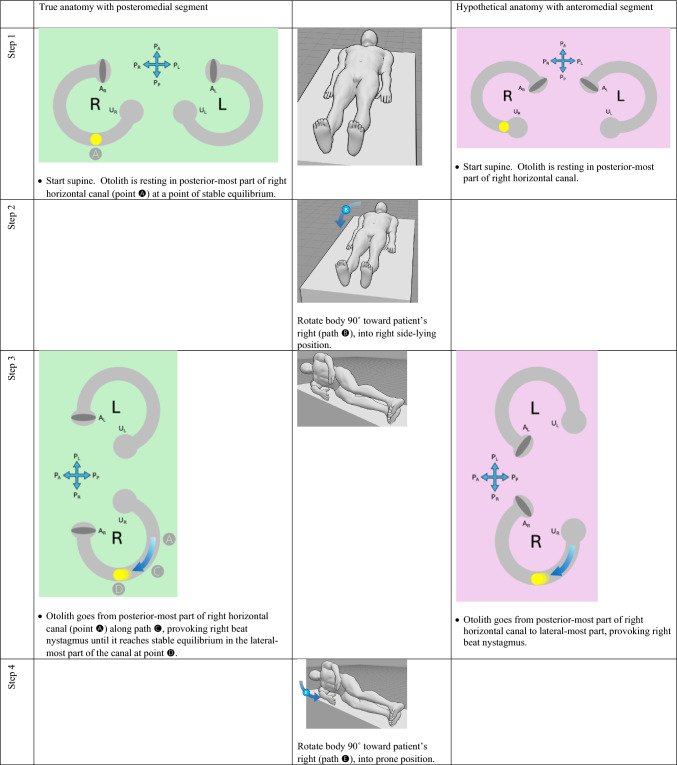

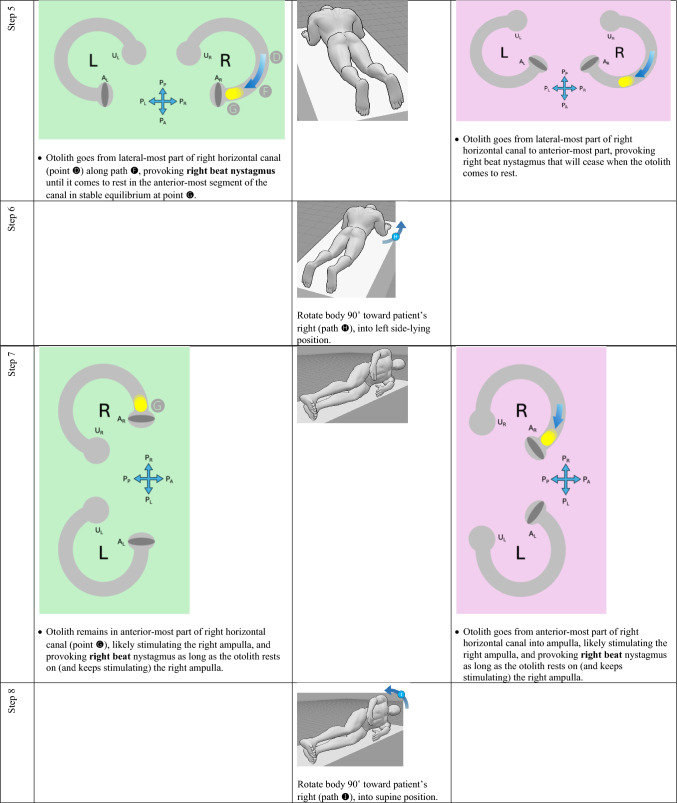

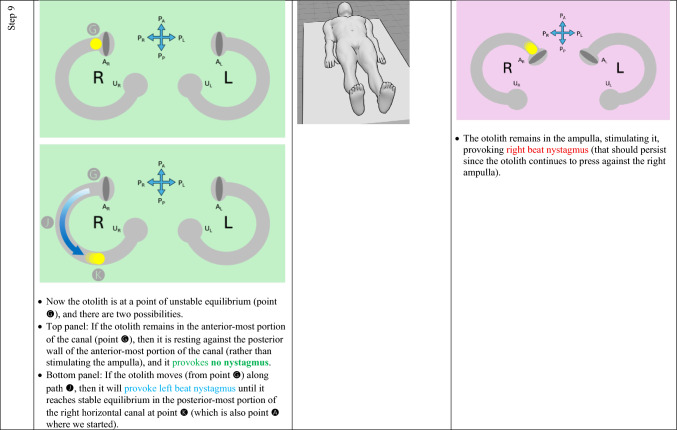
Depiction of the results of rightward roll when the otolith is in the right-sided horizontal canal, comparing true anatomy (with posteromedial segment) and hypothetical anatomy (with anteromedial segment). Abbreviations: R=right; L=left; A_R_=right ampulla; A_L_=left ampulla; U_R_=right utricle; U_L_=left utricle; P_A_=anterior aspect of patient; P_P_=posterior aspect of patient; P_R_=right aspect of patient; P_L_=left aspect of patient.

**Table 2:**
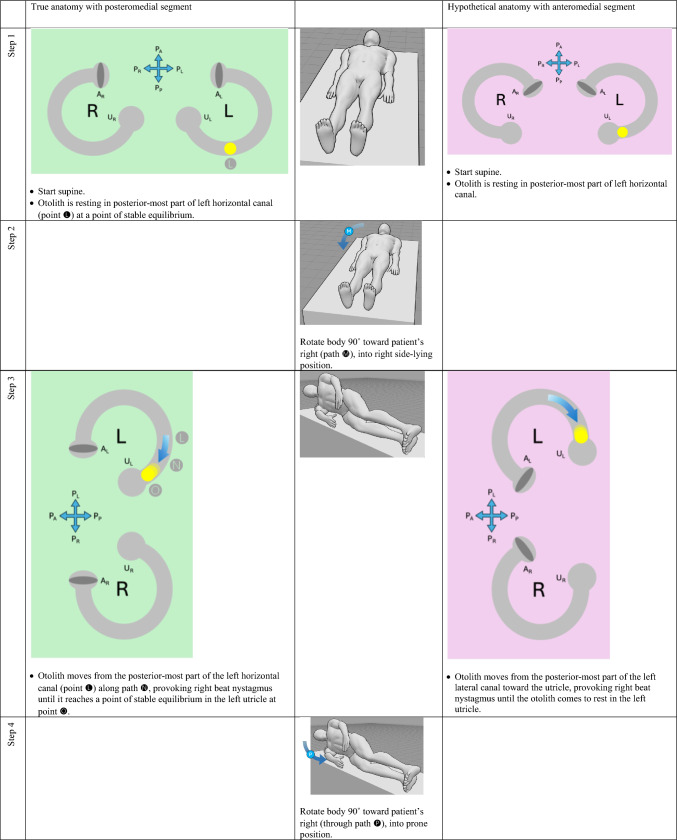

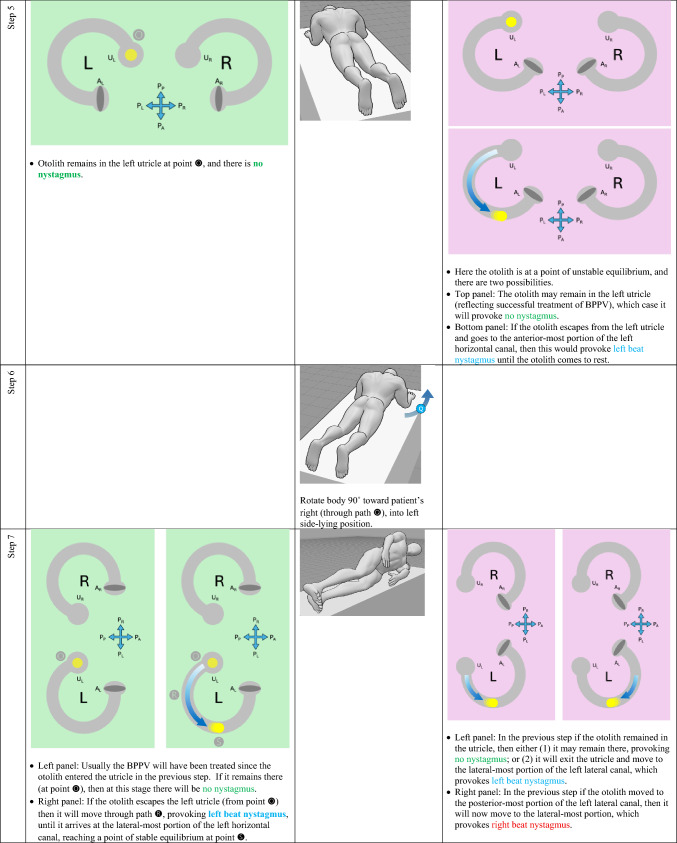

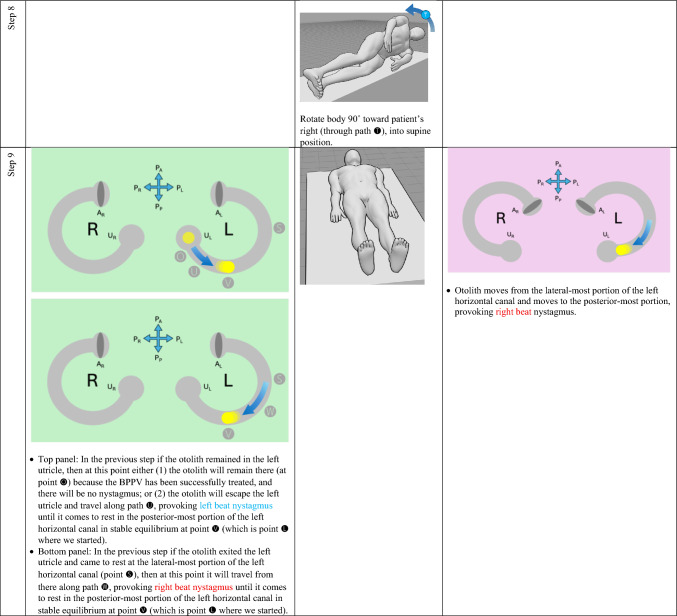
Depiction of the results of rightward roll when the otolith is in the left-sided horizontal canal, comparing true anatomy (with posteromedial segment) and hypothetical anatomy (with anteromedial segment). Abbreviations: R=right; L=left; A_R_=right ampulla; A_L_=left ampulla; U_R_=right utricle; U_L_=left utricle; P_A_=anterior aspect of patient; P_P_=posterior aspect of patient; P_R_=right aspect of patient; P_L_=left aspect of patient.

We summarize the different predictions made by the true anatomy (with a posteromedial segment) and the hypothetical anatomy (with an anteromedial segment) for right-sided horizontal canal BPPV in response to a rightward roll (in Table 3) and leftward roll (in Table 4).

**Table 3:**
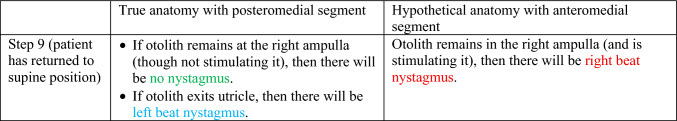
Summary of the different predictions made by the true anatomy (with a posteromedial segment) and the hypothetical anatomy (with an anteromedial segment) for right-sided horizontal canal BPPV in response to a rightward roll

**Table 4:**
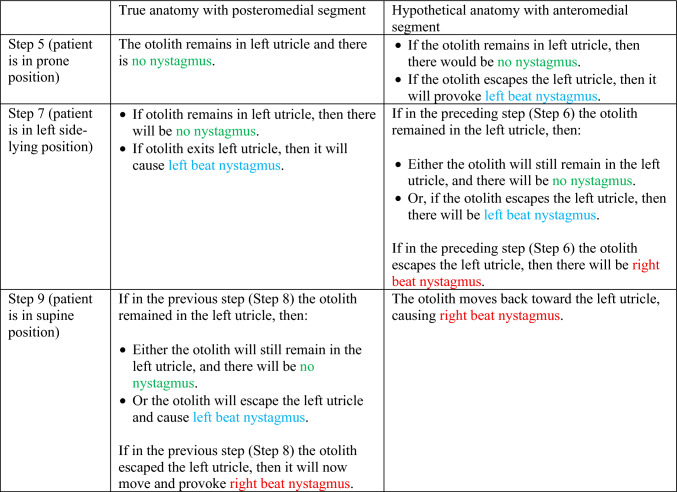
Summary of the different predictions made by the true anatomy (with a posteromedial segment) and the hypothetical anatomy (with an anteromedial segment) for left-sided horizontal canal BPPV in response to a rightward roll

It should be clear from these findings that the true anatomy and the hypothetical anatomy make different predictions about the nystagmus resulting from a roll maneuver.

Focusing on the true anatomy, we now review the effect of a rightward roll when the otolith is in the right-sided versus left-sided horizontal canal in Table 5.

**Table 5:** For true anatomy (with posteromedial segment), a rightward roll maneuver will produce different responses of nystagmus at several steps if the right-sided horizontal canal is affected, versus the left-sided horizontal canal is affected

	Result of rightward turning with otolith in **right** horizontal canal in true anatomy (with posteromedial segment)	Result of rightward turning with otolith in **left** horizontal canal in true anatomy (with posteromedial segment)
Step 5 (patient in prone position)	Right beat nystagmus until otolith comes to rest	No nystagmus because otolith is already in the utricle
Step 7 (patient in left side-lying position)	Right beat nystagmus	Either no nystagmus or left beat nystagmus
Step 7 (patient in supine position)	Either no nystagmus or left beat nystagmus	Either left beat nystagmus or right beat nystagmus

For diagnostic purposes, Step 5 is probably the key position, and reveals that, when turning rightward from supine, through right side-lying, to prone:

- If in the prone position there is right beat nystagmus (which is to say nystagmus whose fast phase is ipsiversive to the direction in which the body had just been turning), then BPPV is affecting the right-sided horizontal canal. This also means that you are in the middle of a logroll maneuver that is treating the incorrect side.
- If in the prone position there is no nystagmus, then the BPPV is affecting the left-sided horizontal canal. This also means that you are in the middle of a logroll maneuver that is treating the correct side.

We believe the above demonstration depicts the predictions clearly, but we also recognize that the format is cumbersome and lengthy. It would be desirable to show this in a more compact format.

## A different data visualization method

We introduce what we believe to be a novel method of data visualization for characterizing the nystagmus of benign paroxysmal positional vertigo (BPPV) as a function of otolith position and direction of gravitational acceleration, applied to the limited case of horizontal canal involvement.

For our purposes, there are two main independent variables to consider:

- *p* = the position of an otolith in degrees within the semicircular canal lumen.
- *d* = direction of gravity in degrees. This assumes that the patient is lying down and the plane of the horizontal canal is perpendicular to the earth.

We will adopt the convention that for both variables:

- 0° (or 360°) points anteriorly (toward the patient’s nose). For the gravitational variable, 0° would occur when the patient is lying prone (face down).
- 90° points toward the patient’s left. For the gravitational variable, 90° would occur when the patient is lying on their left side.
- 180° points posteriorly (toward the patient’s occiput). For the gravitational variable, 180° would occur when the patient is lying supine (face up).
- 270° points toward the patient’s right. For the gravitational variable, 270° would occur when the patient is lying on their right side.

**Fig. 4:**
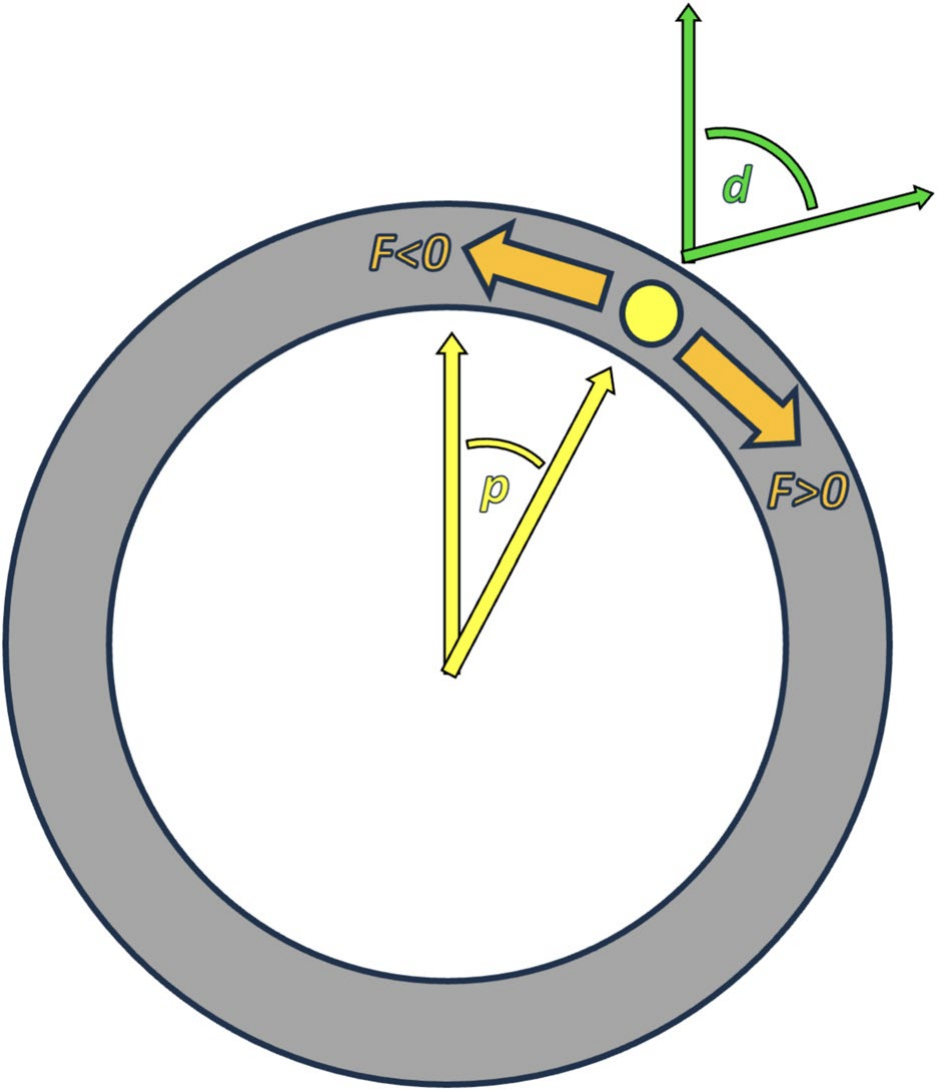
The two variables involved in calculating force on an otolith are: *p* (position of the otolith in the semicircular canal, in degrees) and *d* (the direction of gravity, in degrees)

For configurations in which the position of the otolith and the direction of gravity are not collinear (i.e., differing neither by 0° nor by 180°), as shown in Fig. 4, the resulting force *F* tangential to the lumen would be calculated as the sine of the difference between the two angles (*d* and *p*), as shown in Eq. 1, in which *F* > 0 represents force in the clockwise direction (from the perspective of looking from inferiorly to superiorly, as if observing the patient from the foot of their bed), and *F* < 0 represents force in the counterclockwise direction.

1 $$F=\mathrm{sin} \left(d-p\right)$$

**Fig. 5:**
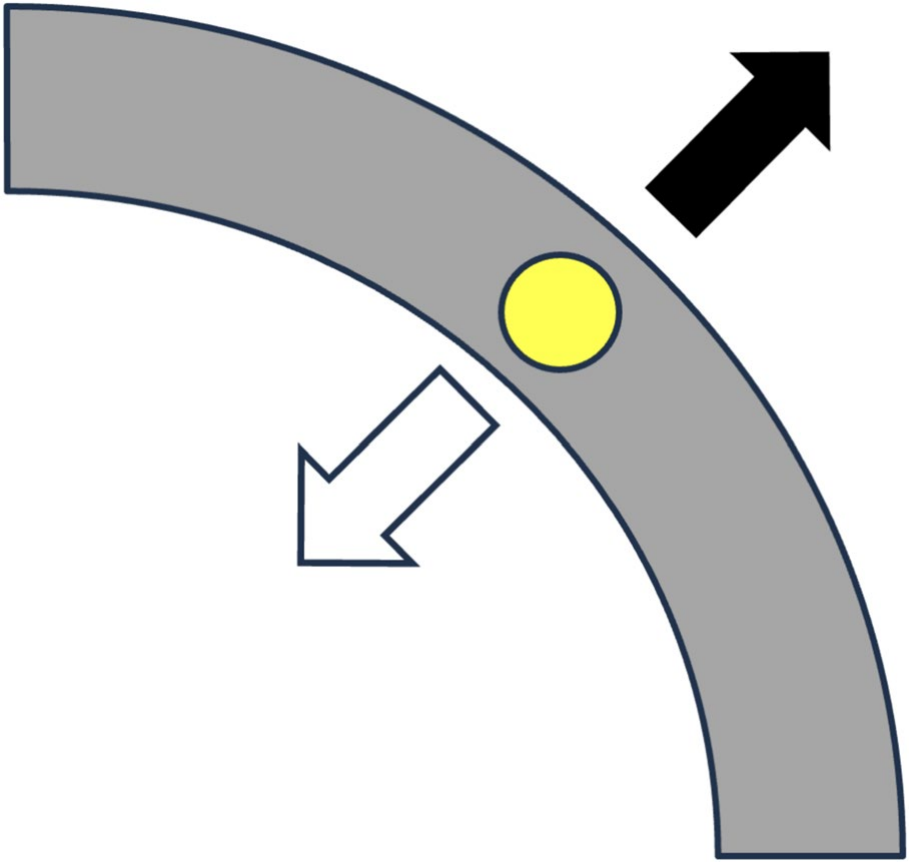
Instances in which forces are purely perpendicular to the tangent at the point in the semicircular canal where the otolith resides. The gray arc represents a segment of the semicircular canal; the yellow circle represents an otolith; the black arrow represents purely centrifugal force (when the direction of gravity and the position of the otolith are equal); the white arrow represents purely centripetal force (when the direction of gravity and the position of the otolith differ by 180°)

Special conditions hold when the force of gravity and the otolith position are collinear. When the position of the otolith and the direction of gravity are equal (i.e., differ by 0°), then the centrifugal force of gravity will maintain the otolith at a point of stable equilibrium; slight force perturbations tangential to the canal lumen will have minimal effect. When the position of the otolith and the direction of gravity are pointing in opposite directions (i.e., differ by 180°), then the centripetal force of gravity will maintain the otolith at a point of unstable equilibrium; but slight force perturbations tangential to the canal lumen will result in acceleration along the canal in the direction of that perturbing force. These situations are illustrated in Fig. 5.

**Fig. 6:**
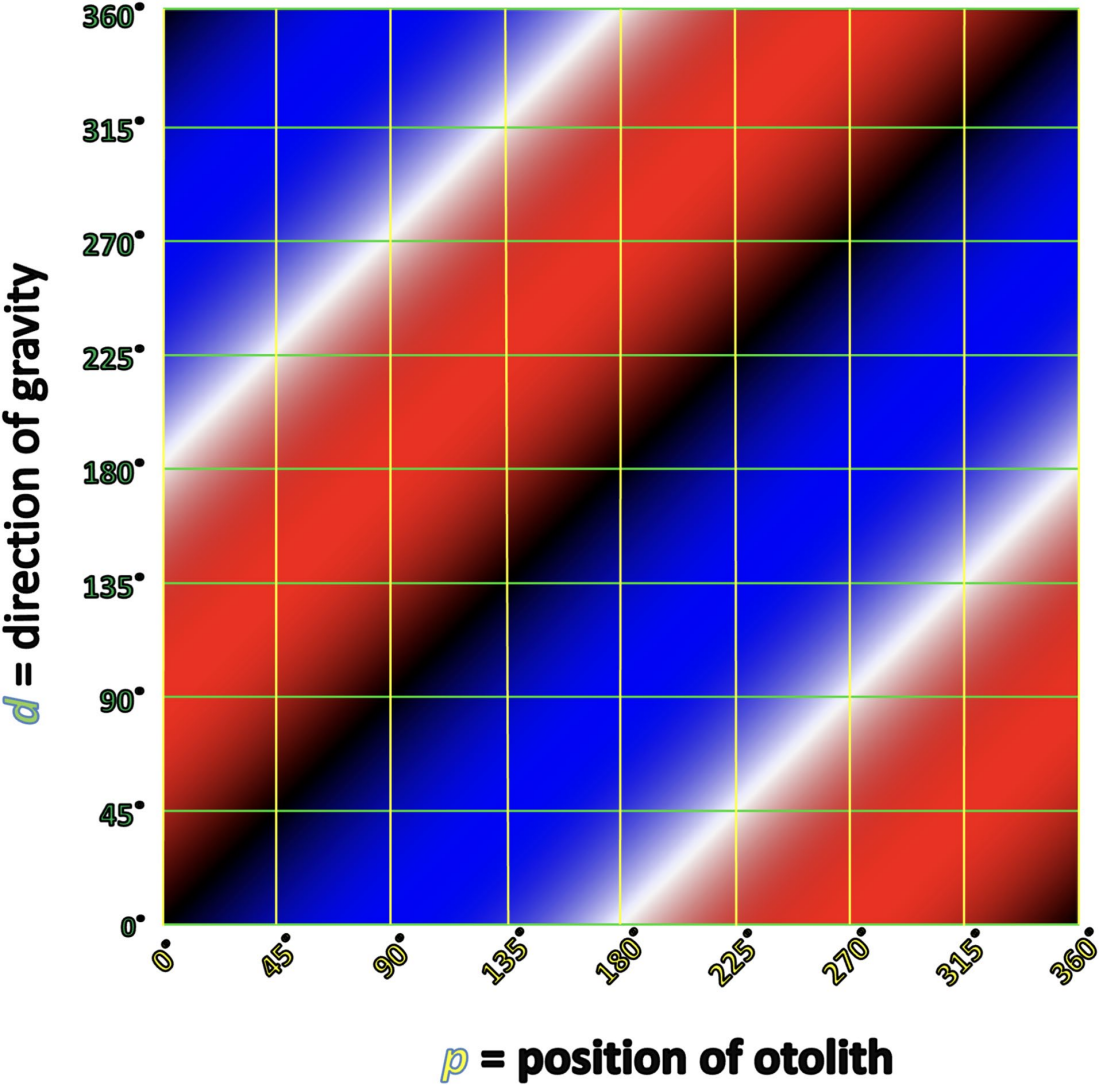
Cartesian plot of otolith position and direction of gravity. The yellow vertical lines represent the position of the otolith (in degrees) along the canal’s lumen. The green horizontal lines represent the direction of gravity (in degrees). For both variables, we adopt the convention that 0° = anteriorly, 90° = toward the patient’s left, 180° = posteriorly, and 270° = toward the patient’s right. The red color represents otolith movement clockwise (when viewing the patient from inferior to superior) that would manifest with right beat nystagmus, the blue color represents otolith movement counterclockwise that would manifest with left beat nystagmus, the saturation of the color represents the magnitude of otolith acceleration (and thus also of its corresponding nystagmus), black represents an area of stable equilibrium, and white represents an area of unstable equilibrium, as discussed in the text

If we iterate Eq. 1 over 360° of otolith positions (*p*) and 360° of gravity directions (*d*), then we get the plot in Fig. 6, in which the red color represents otolith movement clockwise (when viewed from inferior to superior) that would manifest with right beat nystagmus, the blue color represents otolith movement counterclockwise that would manifest with left beat nystagmus, the saturation of the color represents the magnitude of otolith acceleration (and thus also of its corresponding nystagmus), black represents an area of stable equilibrium, and white represents an area of unstable equilibrium.

**Fig. 7:**
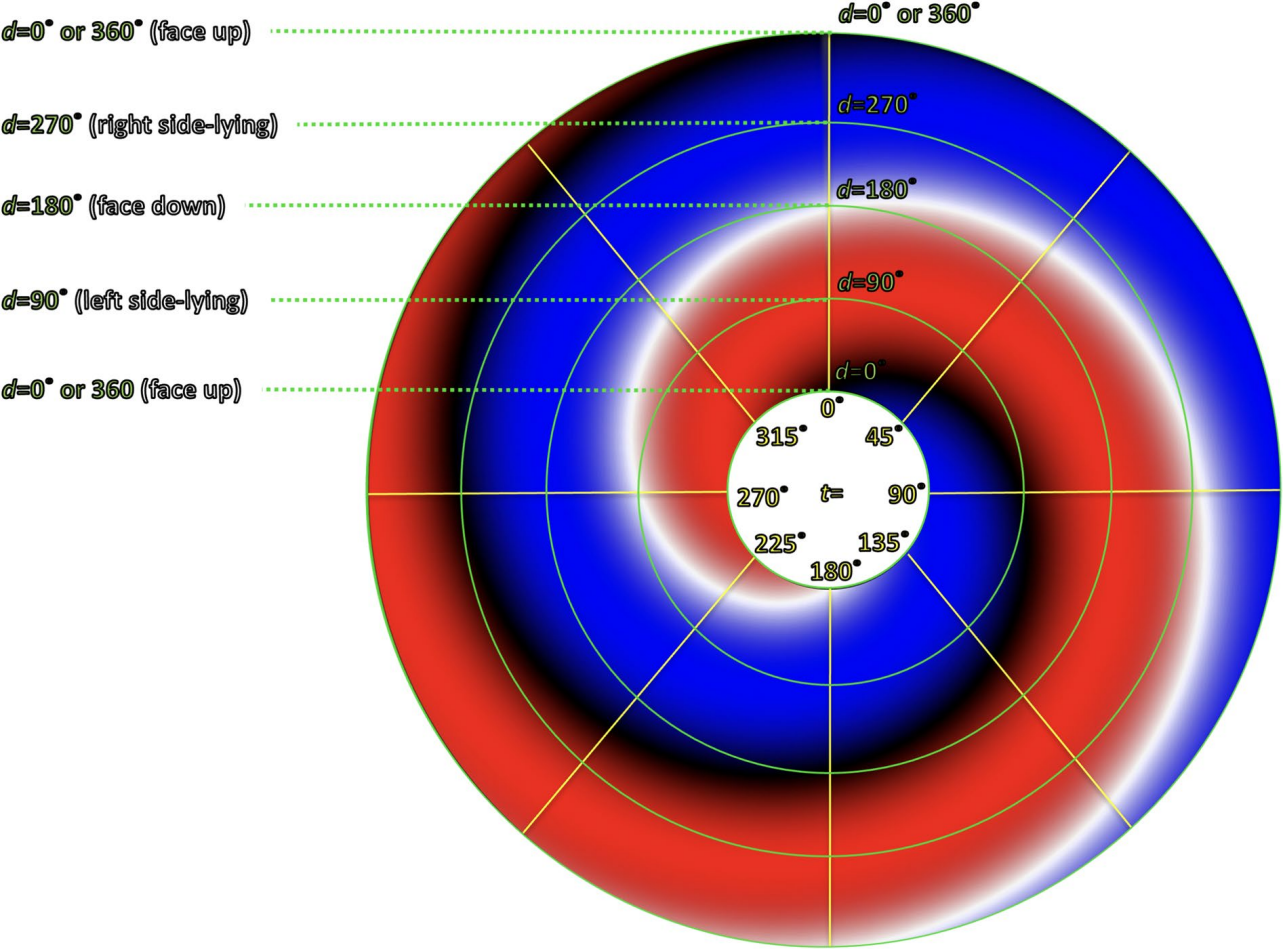
Polar coordinate plot of otolith position and direction of gravity. The radial axes (yellow) represent p (position of otolith in the semicircular canal, in degrees). The circular axes (green) represent d (direction of gravity, in degrees). The remaining colors (red, blue, black, white) have the same significance as in the corresponding Cartesian figure

Some readers may find it more intuitive to transform this from Cartesian to polar coordinates, as shown in Fig. 7. The resulting pattern has the appearance of a “grand design spiral galaxy.” Despite the different appearance, the polar plot in Fig. 7 would reflect the same data as the Cartesian plot in Fig. 6.

**Fig. 8:**
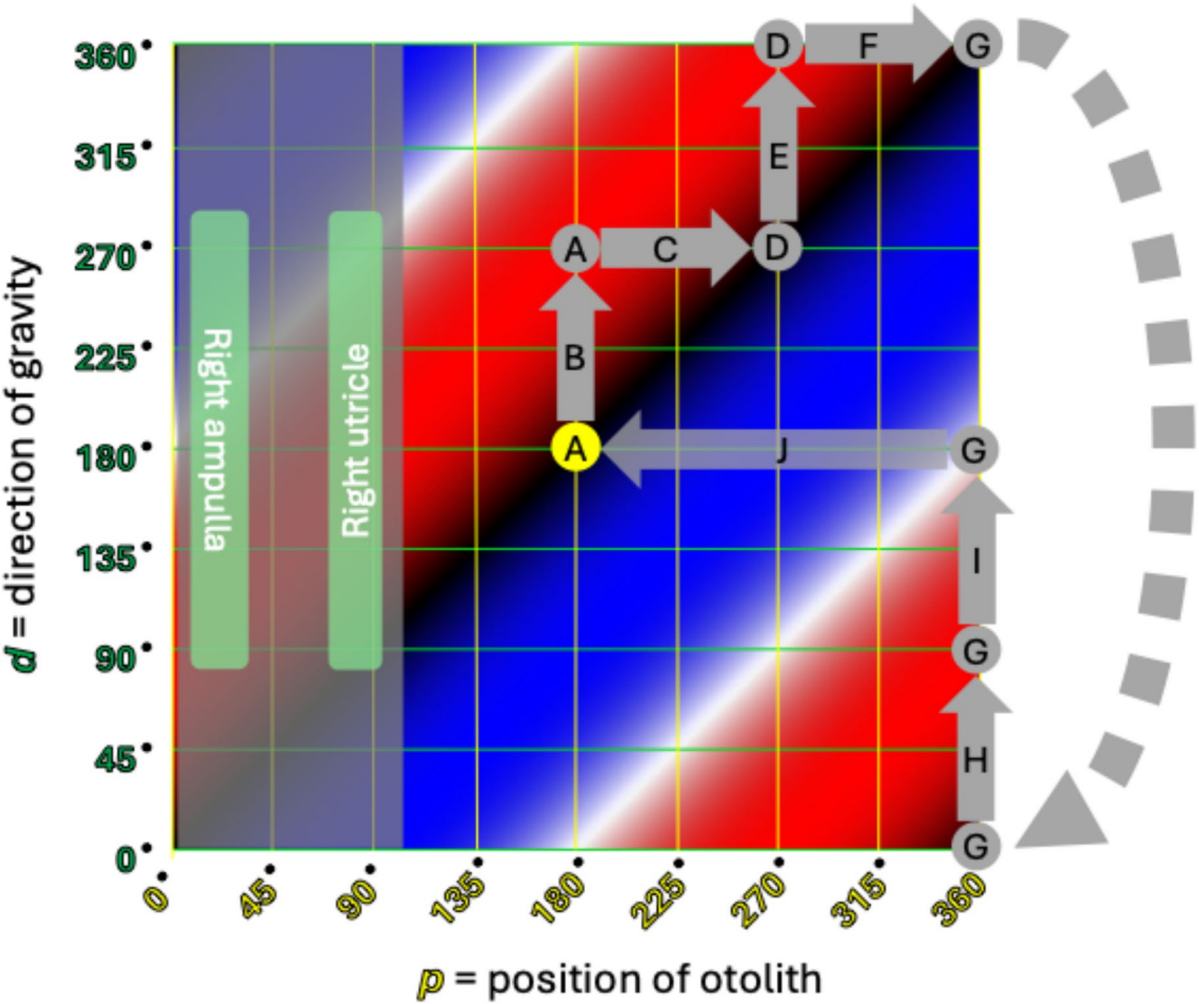
Cartesian plot of what happens in right-sided horizontal canal BPPV when the patient begins in a supine position and rolls rightward. The letters correspond to the positions and paths described in Table 1. The significance of the colors is described in the text and in the caption to Fig. 6. See text for details

**Fig. 9:**
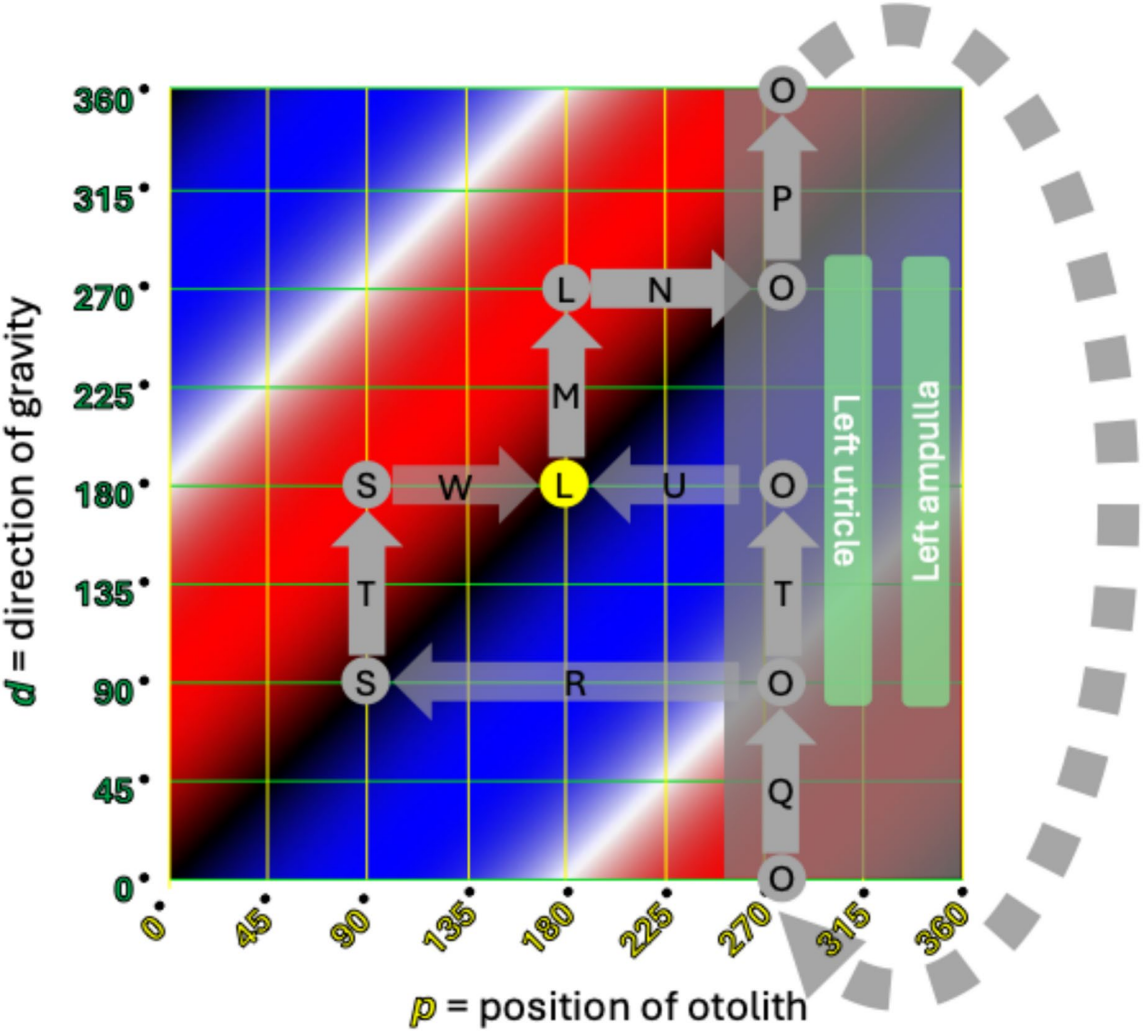
Cartesian plot of what happens in left-sided horizontal canal BPPV when the patient begins in a supine position and rolls rightward. The letters correspond to the positions and paths described in Table 2. The significance of the colors is described in the text and in the caption to Fig. 6. See text for details

The Cartesian configuration is easier to plot, so we shall use that here. With the Cartesian plot, using the letters ( through ) in Tables 1 and 2, we map out the same series of events that occur for right-sided horizontal canal BPPV with rightward roll for right-sided horizontal canal BPPV in Fig. 8, and left-sided horizontal canal BPPV in Fig. 9. The dotted gray pathway reflects that 0° = 360°, so progressing upward from the top of the plot will “loop” back down to the bottom of the plot. The translucent gray arrows reflect those points in the sequence where there is more than one possible outcome. For example, the translucent path  reflects what may happen when the otolith exits (versus does not exit) a point of unstable equilibrium; the translucent paths , and reflect what may happen when the otolith escapes (versus does not escape) the utricle.

The data plotted in Figs. 8 and 9 take the same body positions and positional changes (starting supine and rolling rightward), and depict the resulting changes in otolith position and elicited nystagmus.

Points and paths  through  in Fig. 8 correspond to points and paths  through  in Fig. 9. These demonstrate the key step (Step 5) described in Table 5. Specifically:

- Fig. 8 shows that from the right side-lying position at point  (*d* = 270°), rolling rightward (positional change ) to supine (*d* = 180°), subsequently elicits right beat nystagmus (path , which moves through red) until the otolith reaches a point of stable equilibrium (black) at point .
- Fig. 9 shows that the right side-lying position at point  (*d* = 270°) the otolith is already in the left utricle, so rolling rightward (positional change ) to supine (*d* = 180°) elicits no nystagmus (there are no paths along the horizontal axis, and thus no otolith movement to elicit any nystagmus).

Thus, the information in Figs. 8 and 9 compactly convey all the information whose exposition in Tables 1 and 2, respectively, is much longer.

## Limitations

We pointed out that the patterns of nystagmus at Step 5 (in Tables 1, 2**,** 5) help lateralize the affected horizontal canal. In this step the patient is prone, and in such a downward-facing position, it would be difficult for an examiner to observe nystagmus merely on face-to-face examination. Practically, this may only be discernible with an instrumented ocular motor examination (using infrared video Frenzel goggles) which is not available to all clinicians, and thereby limits the applicability of this method.

## Summary and conclusions

Horizontal canal benign paroxysmal positional vertigo poses a particular diagnostic challenge in terms of lateralization. Some of the literature based on the “bow and lean” test assumes a hypothetical anatomical configuration in which the horizontal canal has an anteromedial segment. The true anatomy has a posteromedial segment. We show that the hypothetical anatomical configuration and the true anatomical configuration make different predictions about the nystagmus elicited by a roll maneuver.

We further show that the true anatomical model predicts that for a roll maneuver that begins in the supine position, going from the side-lying position to the prone position should generate different patterns of nystagmus depending on which side is affected. This has implications for treatment, since selection of an appropriately targeted therapeutic maneuver depends on which side is affected.

We conclude by offering a data visualization approach that compactly depicts these findings.
